# Targeting Glucosylceramide Synthase: Innovative Drug Repurposing Strategies for Lysosomal Diseases

**DOI:** 10.3390/ijms26052195

**Published:** 2025-02-28

**Authors:** Giorgia Canini, Elena Mazzinelli, Giuseppina Nocca, Wanda Lattanzi, Alessandro Arcovito

**Affiliations:** 1 Dipartimento di Scienze Biotecnologiche di Base, Cliniche Intensivologiche e Perioperatorie, Università Cattolica del Sacro Cuore, Largo Francesco Vito 1, 00168 Rome, Italy; giorgia.canini@unicatt.it (G.C.); elena.mazzinelli@unicatt.it (E.M.); giuseppina.nocca@unicatt.it (G.N.); 2 Fondazione Policlinico Universitario “A. Gemelli”, IRCCS, Largo A. Gemelli 8, 00168 Rome, Italy; wanda.lattanzi@unicatt.it; 3 Department of Life Science and Public Health, Università Cattolica del Sacro Cuore, Largo Francesco Vito 1, 00168 Rome, Italy

**Keywords:** lysosomal storage diseases, sphingolipidoses, drug repurposing, cross-docking, virtual screening, molecular dynamics simulations

## Abstract

Sphingolipidoses, a subgroup of lysosomal storage diseases (LSDs), are rare and debilitating disorders caused by defects in sphingolipid metabolism. Despite advancements in treatment, therapeutic options remain limited. Miglustat, a glucosylceramide synthase EC 2.4.1.80 (GCS) inhibitor, is one of the few available pharmacological treatments; however, it is associated with significant adverse effects that impact patients’ quality of life. Drug repurposing offers a promising strategy to identify new therapeutic agents from approved drugs, expanding treatment options for rare diseases with limited therapeutic alternatives. This study aims to identify potential alternative inhibitors of GCS through a drug-repurposing approach, using computational and experimental methods to assess their therapeutic potential for sphingolipidoses. A library of approved drugs was screened using advanced computational techniques, including molecular docking, molecular dynamics simulations, and metadynamics, to identify potential GCS inhibitors. Promising candidates were selected for further in vitro validation to evaluate their inhibitory activity and potential as therapeutic alternatives to Miglustat. Computational screening identified several potential GCS inhibitors, with Dapagliflozin emerging as the most promising candidate. Experimental validation confirmed its efficacy, revealing a complementary mechanism of action to Miglustat while potentially offering a more favorable side effect profile. This study underscores the utility of computational and experimental methodologies in drug repurposing for rare diseases. The identification of Dapagliflozin as a potential GCS inhibitor provides a foundation for further preclinical and clinical evaluation, supporting its potential application in the treatment of sphingolipidoses.

## 1. Introduction

Lysosomal storage diseases (LSDs) represent a diverse group of over 70 rare metabolic disorders characterized by lysosomal dysfunction, most inherited in an autosomal recessive pattern. Although individually rare, these conditions collectively affect approximately 1 in 5000 live births [1,2,3]. Their progression often involves neurodegeneration and can impact multiple organ systems, including the brain, liver, spleen, and heart. Symptoms span from severe to milder forms, with the severe cases often presenting early-onset symptoms that lead to premature death, while the milder cases are marked by later-onset variants. There is notable variability in the onset, severity, and progression of the diseases [4]. LSDs are caused by deficiencies in lysosomal acid hydrolases enzymes, essential for breaking down macromolecules into smaller, recyclable components, or by other deficiencies associated with ineffective lipid trafficking [5,6,7,8]. When these enzymes are deficient or non-functional, substrate accumulation within lysosomes disrupts cellular function, leading to apoptosis and the enlargement or dysfunction of affected organs [4,9,10,11]. Sphingolipidoses are a well-known subgroup of LSDs caused by disruptions in sphingolipid metabolism, and include Gaucher disease, Fabry disease, and Niemann–Pick type C (NPC) [12,13,14,15,16]. Gaucher disease is the most prevalent type, resulting from glucocerebrosidase deficiency; Fabry [13,14,15,16,17] and NPC [4,18] are instead linked to the accumulation of glucosylceramide-derived products. Developing novel pharmacological therapies for rare diseases like sphingolipidoses poses significant challenges due to limited patient populations, high costs, and restricted treatment options. Despite significant advances in the management of these rare diseases, the availability of effective and safe treatments is still limited. Treatments for sphingolipidoses related to LSDs are based on two main concepts: reducing the concentration of accumulated substrates or slowing down their synthesis rate. Current therapeutic approaches include bone marrow transplantation (BMT) [19,20,21], enzyme replacement therapy (ERT) [22,23], substrate reduction therapy (SRT) [24,25,26], chaperone therapy [27,28,29], and supportive care [30]. Among these, ERT is considered the current standard of care for sphingolipidoses, as most approved drugs rely on this type of treatment. However, a significant limitation of ERT lies in its reduced effectiveness in treating sphingolipidoses that affect the central and peripheral nervous systems, representing a considerable barrier to its broader application [30,31,32]. To overcome this limitation, alternative approaches such as gene therapy (GT) have emerged, offering innovative methods with the potential to address the underlying genetic causes of sphingolipidoses. GT represents a promising frontier but is not without challenges: high costs and the need for extensive research and safety evaluations remain significant barriers. From a therapeutic standpoint, the availability of diverse treatment options has been instrumental in combating these debilitating diseases, providing benefits to an increasing number of patients [30]. Combinatorial therapeutic strategies are currently employed to enhance treatment efficacy, alleviate symptoms, and improve quality of life. In addition to traditional approaches, drug repurposing has emerged as a particularly promising strategy for rare diseases, including LSDs. By identifying new applications for existing drugs, drug repurposing offers a faster, cost-effective path to therapeutic development, potentially accelerating patient access to effective treatments compared to the traditional drug design and development process. Therefore, it could play a pivotal role in addressing the unmet needs of patients with sphingolipidoses, expanding therapeutic opportunities, and improving outcomes for these debilitating disorders [33,34,35].

In this work, we selected glucosylceramide synthase EC 2.4.1.80 (GCS) as the possible target, as it is inhibited by Miglustat, a drug approved for the treatment of Gaucher disease [36,37] and NPC in patients with mild to moderate neurological symptoms. Miglustat received approval in Europe in November 2002 for mild to moderate Gaucher disease type 1 [37] and, in 2009, for the treatment of neurological symptoms of NPC in several countries [38]. More recently, it was approved by the U.S. Food and Drug Administration for use in combination with Arimoclomol, an inhibitor of the organic cation transporter 2 (OCT2) [39]. This therapeutic protocol presents some side effects, such as urticaria and angioedema, which have been reported in some cases, and has some common adverse effects such as upper respiratory tract infection, diarrhea, and decreased weight. Using a drug-repurposing approach, we propose a possible alternative to Miglustat, thus widening the possibility for patients suffering this pathological condition. Our approach combines computational and experimental methodologies, starting with the characterization through molecular dynamics (MD) and metadynamics simulations of the complex GCS with Miglustat, its approved inhibitor, to establish a further baseline for drug repurposing. A subset of five protein conformations derived from the inhibited state was selected and combined with an additional subset of five conformations each from the *apo* and *holo* states, previously identified [40]. These conformations were used for a virtual screening of a database of approved drugs from ZINC15 [41]. The screening process integrated multiple computational approaches, including cross-docking [42,43], molecular docking-based virtual screening [42,43,44], molecular mechanics/generalized born surface area (MM-GBSA), free energy calculations [45,46], and MD simulations, to identify potential novel inhibitors. Initially, cross-docking was performed to generate docking grids for all receptor conformations, followed by docking of all ligands to each receptor structure. A threshold of approximately −6 kcal/mol was applied to filter the docking results. Virtual screening was then carried out by re-docking selected compounds to refine and validate the initial cross-docking results. To assess the pharmacokinetic and drug-likeness properties of the top-ranked compounds, key ADME parameters were calculated, including predicted apparent Caco-2 cell permeability (QPPCaco), estimating the predicted brain–blood partition coefficient (QPlogBB), and compliance with Lipinski’s rule of five. For a more detailed evaluation of binding affinity, MM-GBSA calculations were performed to estimate the free binding energies of each protein–ligand complex. Finally, MD simulations were conducted to analyze the stability of the selected enzyme–ligand complexes over time. This in silico step was followed by in vitro validation in a cellular model, to confirm the findings through the comparison of the cell viability in the presence of these selected compounds with the Miglustat. This integrated strategy will be carefully described in this work as it aims to expand therapeutic options for sphingolipidoses by identifying a new repurposed pharmacological alternative.

## 2. Results and Discussion

A previous computational characterization of the GCS enzyme, in the presence of the manganese ion (Mn^2+^) cofactor and both endogenous substrates, UDP-glucose and ceramide [40,47,48], was addressed to clearly identify the mechanism of enzymatic activity of GCS that was in perfect agreement with the experimental results [40]. In this work, we focused our attention on the interaction of GCS with the approved Miglustat inhibitor as a starting point for a further drug repurposing approach.

### 2.1. Induced Fit Docking for Miglustat: A Unique Treatment for NPC as a GCS Inhibitor

**Figure 1:**
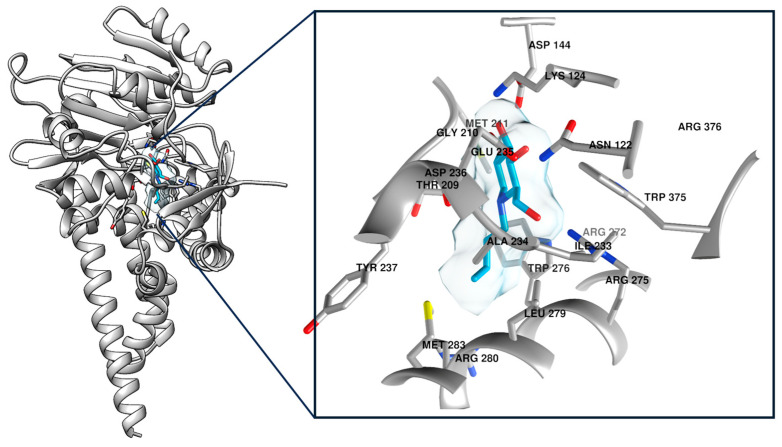
The figure illustrates the enzyme–inhibitor complex, with a detailed close-up highlighting the key interactions within a 5 Å radius of the inhibitor (showed in cyan). This binding pose, identified as the most favorable result from the induced fit docking protocol, achieved a docking score of −7.349. The close-up reveals critical residues in the enzyme’s active site that contribute to the stability and specificity of the inhibitor binding.

Following the protocol outlined in the Materials and Methods section, induced fit docking of the inhibitor with the GCS structure using the Maestro Schrödinger software suite (v. 2020-3) was performed. This approach optimized the receptor–inhibitor interactions to account for potential conformational flexibility within the GCS binding site. As a result, 18 distinct docking poses were generated. The top-ranked pose, exhibiting a docking score of −7.349, was selected for further analysis based on its predicted stability and favorable binding interactions with the target (Figure 1). This choice was guided by the docking score and a detailed visual inspection of the binding interface.

**Figure 2:**
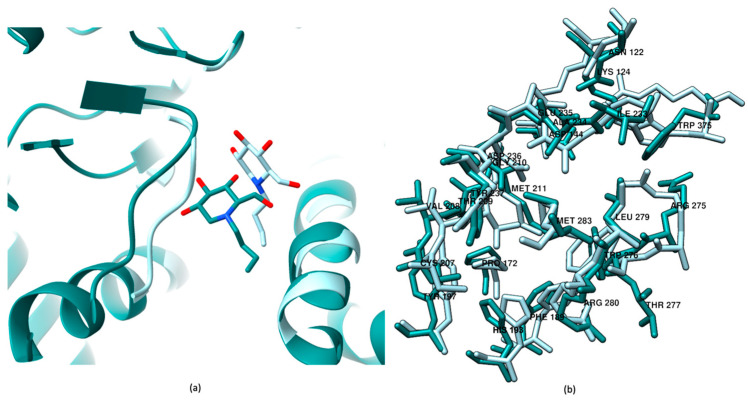
Comparison of the system between the best pose obtained from induced fit docking protocol and the binding after cluster analysis of the last 500 ns of the MD simulation. (**a**) The most representative structure from the MD simulation clustering is shown in teal, with the best pose from the induced fit docking procedure depicted in light cyan. (**b**) Using the same color code, the amino acids within 5 Å of the inhibitor are displayed, revealing shifts in key residues such as Arg 275, Trp 375, His 193, Trp 276, Asp 144, Pro 172, and Tyr 197.

To further explore the enzyme’s binding interactions with the inhibitor, a 1 µs MD simulation was performed. Clustering analysis was applied to the final 500 ns of the simulation to identify the most representative conformations of the GCS–inhibitor complex (Figure S1A,B). The results, illustrated in Figure 2a, show the final equilibrated position of Miglustat in teal, allowing a direct comparison with its initial docking pose, represented in light cyan. This visual comparison reveals shifts in the inhibitor’s position and orientation during the MD simulation, providing a dynamic and more detailed understanding of Miglustat’s interaction with the GCS active site. Figure 2b displays the orientations of amino acids within 5 Å of the inhibitor, with significant rearrangements observed in key residues [40,49], such as Arg275, Trp375, His193, Trp276, Asp144, Pro172, and Tyr197, highlighting the adaptability of the binding interface over time.

**Figure 3:**
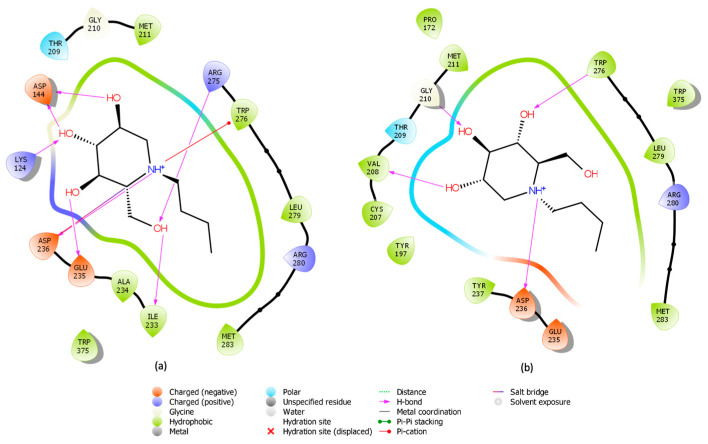
Comparison of Miglustat interactions analyzed using the Maestro Schrödinger software ligand interaction tools. (**a**) Interactions after induced fit docking. (**b**) Interactions based on the clustering analysis of the final 500 ns of the MD simulation.

The change in Miglustat’s orientation results in distinct interaction profiles between the inhibitor and GCS when comparing the induced fit docking results (Figure 3a) with the most representative structure from the final 500 ns of the MD simulation. This comparison highlights two different sets of interactions with GCS residues. In Figure 3a, Miglustat forms multiple hydrogen bonds (H-bonds) with Asp144, Asp236, Glu235, and Arg275, including a notable salt bridge with Asp236. In contrast, Figure 3b, it reveals a different interaction pattern, where H-bonds are formed with Val208, Gly210, Trp276, and Asp236. These residues are key players in the SNi-type reaction mechanism (substitution nucleophilic internal reaction mechanism), as studied in precedent work [40], by ensuring proper substrate orientation.

The observed changes suggest that the ligand has undergone a conformational shift, altering its interactions with critical residues in the GCS active site. These modifications may significantly affect the ligand’s stability and its precise positioning within the binding site. To explore this further, a well-tempered funnel metadynamics (FM) study was conducted to determine the optimal orientation and binding interactions of the inhibitor within the GCS active site.

### 2.2. Well-Tempered FM Analysis of the GCS–Inhibitor Complex

The well-tempered FM was performed using, as the starting point for the simulation, the most stable pose obtained from the clustering analysis of the preceding MD simulation. To explore the conformational landscape of the active site in detail, three collective variables (CVs) were selected for the FM simulation. CV1 represents the position along the rotational axis of the funnel-shaped active site (reported in Supplementary Materials Figure S2). CV2 represents the radial distance from this axis and CV3, the distance between the carbon atom of the carboxyl group in Asp236 and the nitrogen atom in Miglustat’s piperidine ring (d_CG-N_). To ensure system stability during the FM simulation, positional restraints were applied to the enzyme’s alpha-carbons of the protein backbone (Cα atoms). The simulation, which ran for 6 µs and 280 ns, provided a comprehensive view of the inhibitor’s interactions with active site residues and detected the optimal positioning of Miglustat for effective GCS inhibition. The Gaussian heights gradually approached zero, indicating that the well-tempered FM simulation converged after 6 µs, as illustrated in the energy landscape reported in Supplementary Materials (Figure S3A). This convergence demonstrates that the simulation successfully explored the relevant conformational space. Additionally, Figure S3B shows the ligand repeatedly exiting and re-entering the entrance channel. This movement allowed the inhibitor to stabilize within the GCS binding site, with its piperidine ring consistently aligning as the polar head of ceramide. The precise moment when Miglustat occupied the ceramide binding site is marked by a red dashed line in the same figure.

**Figure 4:**
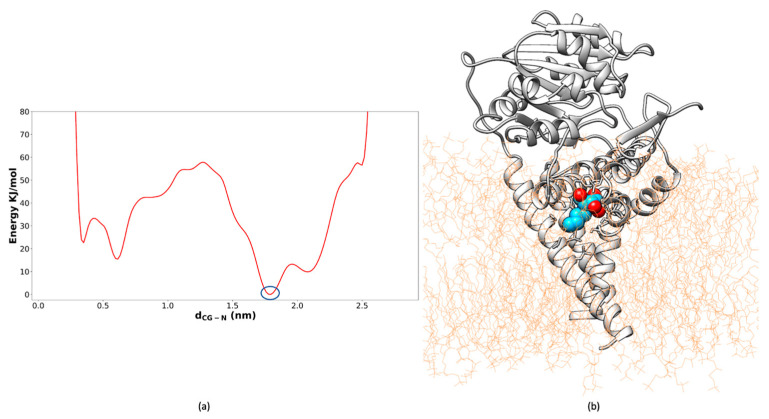
FM analysis. (**a**) Energy profile of the simulation reporting the absolute minimum of energy (blue circle) in function of the CV3 (d_CG-N_), specifically at distance of 1.77 nm between the two chosen atoms. (**b**) Miglustat (displayed in sphere representation, with carbon atoms in cyan and oxygen atoms in red) in its most energetically favorable state, showing a specific orientation to replace the ceramide, as studied in previous work [40].

The absolute minimum energy state of the inhibitor within the ceramide binding site was successfully determined as reported in Figure 4a. The FM simulation indicated that this Miglustat conformation, showed in Figure 4b, is the most energetically favorable.

**Figure 5:**
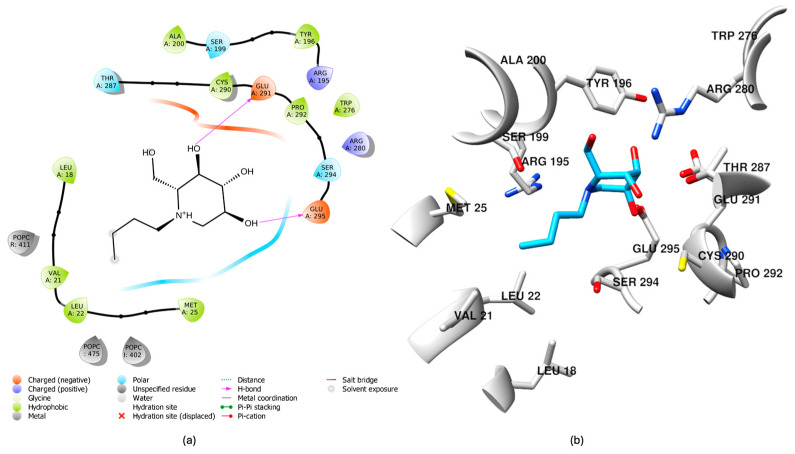
FM analysis. (**a**) Miglustat representation in binding site, identified as having the lowest energy. (**b**) Miglustat interactions analyzed using the Maestro Schrödinger software ligand interaction tools, displaying inhibitor–receptor interactions including hydrophobic contacts, H-bonds, salt bridges, π–cation interactions, π-stacking, water bridges. This result derives from the analyses of the stable conformation obtained with the well-tempered FM simulation.

After 1901 ns of simulation, the inhibitor achieved a state that clearly favored a specific orientation within the binding pocket. This orientation is characterized by a stable conformation and significant interactions with key target residues, suggesting a high binding affinity. The inhibitor engages in interactions with critical amino acids, such as Arg280 and Trp276, which have been previously identified as important for interacting with UDP-glucose [40]. Furthermore, H-bonds are formed with Glu291 and Glu295, which assist in the proper orientation of the inhibitor within the binding site (showed in Figure 5a with ligand-interaction visualization in Maestro Schrödinger software). These results imply that both H-bonds and hydrophobic interactions are crucial for stabilizing the GCS–inhibitor complex. Collectively, this analysis demonstrates that Miglustat acts as a ceramide analog [50], shown in Figure 5b, occupying the binding site in a way that prevents the proper positioning of ceramide, an essential step in the glycosylation reaction [51].

This result is significant as it validates the findings of T. D. Butters et al. [51], demonstrating that Miglustat interferes with glycosphingolipid biosynthesis by specifically targeting ceramide binding at the enzyme’s active site, without affecting UDP-glucose binding. Therefore, Miglustat acts as a competitive inhibitor of ceramide while remaining non-competitive with UDP-glucose [51].

### 2.3. In Silico Drug Repurposing

In the initial phase of the drug repurposing protocol, a comprehensive selection of enzyme structures was performed to conduct cross-docking studies. To establish a robust and reliable methodology, a total of fifteen distinct conformations of the GCS enzyme were chosen, representing various structural states. Specifically, five structures were obtained from clustering analysis of MD simulations for the *apo* form of GCS, simulation and analysis performed in the previous work [40], providing insights into the unbound state of the enzyme. Another five structures were derived from well-tempered FM simulations of the GCS enzyme in complex with its substrates, representing different *holo* conformations that promote ceramide glycosylation (when d_C1-Oc_ is at or near its minimum) [40]. Additionally, five structures were acquired from clustering analysis of MD simulations involving Miglustat, as previously described. With this different selection of the conformations of the GCS enzymes we tried to improve the reliability of the cross-docking results. In the preliminary phase of protocol development, Glide SP docking was utilized to screen a library of 5903 compounds from the ZINC15 [41] subset, which includes FDA-approved drugs and those listed in DrugBank [52]. This library was evaluated against 15 distinct conformational states of GCS. Cross-docking was conducted to generate grids for all receptor conformations and to dock each ligand to every receptor structure. A uniform docking score threshold of −6 was applied to select compounds for the subsequent Glide XP docking phase. The number of successfully docked ligands for each receptor conformation after the Glide XP protocol is summarized in Supplementary Materials Table S1. Following the cross-docking phase, it is crucial to perform further screening to reduce the number of candidate drugs and focus on those with the most promising results. While cross-docking produces a large dataset, not all structures and complexes are relevant for further analysis. Through this screening process, only those structures with the highest docking scores and the most favorable GCS–ligand interactions are considered. This step is essential to enhance the efficiency of the drug repurposing process and ensure that only the most promising candidates are considered for further development and optimization.

### 2.4. Virtual Screening Protocol

The next phase of the virtual screening (VS) workflow involved re-docking to refine and validate the initial cross-docking results. The goal of this step was to confirm the binding conformations and interactions of the selected compounds, thereby enhancing the accuracy of binding affinity predictions and ensuring the reliability of identified inhibitors for further experimental validation. This approach provided a thorough evaluation of potential drug candidates across different enzyme conformations, enabling the identification of promising inhibitors for subsequent studies. After visual inspection and applying a docking score threshold of −7.0, a subset of molecules was selected. XP re-docking was then conducted on this subset with each receptor conformation. Table S2, in Supplementary Materials, summarizes the number of ligands selected from cross-docking and those successfully docked. These ligands were further analyzed using the QikProp module to assess their physicochemical properties and ADME profiles, followed by evaluation based on Lipinski’s rule of five. For the top eleven compounds, docking scores ranged from −11.769 to −9.142 kcal/mol. Notably, most of these compounds (eight out of eleven) formed H-bonds with Asp236, while only three formed H-bonds with His193, a residue critical for inhibitor binding [53]. Additionally, the majority (seven out of eleven) exhibited π-stacking interactions with Trp276. These interactions are significant, as these residues have been identified as crucial for the catalytic activity of the GCS enzyme [40]. The results suggest that targeting these specific interactions may be key to developing potent GCS inhibitors. All findings are presented in Table 1.

Table S3 presents the ADME predictions for the eleven compounds, with a focus on their permeability metrics as determined by QPPCaco in nm/s, QPlogBB, and by predicted octanol/water partition coefficient (QPlogPo/w). The results indicate that three compounds, Nebivolol, Canagliflozin, and Carvedilol, exhibit favorable oral absorption and moderate permeability to the central nervous system (CNS). Similarly, three other compounds, Dapagliflozin, Ertugliflozin, and Floctafenine, show acceptable oral absorption and moderate CNS penetration. In contrast, most of the remaining compounds, with the exception of Benazepril, which demonstrates moderate CNS penetration, show unsatisfactory values for both QPPCaco and QPlogBB.

None of the analyzed compounds violated Lipinski’s rules regarding molecular weight, H-bond donors, or H-bond acceptors. However, one ligand, Macimorelin, while not violating Lipinski’s criteria, is flagged as high risk due to its high polar surface area (PSA) of 165.171 Å^2^ and the presence of 10 rotatable bonds (Table 2).

Given its poor performance in the ADME analysis, Macimorelin has been excluded from further consideration.

Based on the ADME predictions from QikProp and the evaluation of Lipinski’s rule compliance, we can anticipate the permeability of the remaining compounds through QPPCaco and QPlogBB values (Table S3). Nebivolol, Canagliflozin, and Carvedilol generally show favorable values for oral absorption and moderate CNS permeability. Dapagliflozin, Ertugliflozin, and Floctafenine also demonstrate acceptable oral absorption and moderate CNS penetration. Benazepril stands out with moderate CNS penetration and a QPlogPo/w value of 1.7, indicating a favorable balance of lipophilicity and hydrophilicity, suggesting good bioavailability and effective drug delivery. However, the other compounds exhibit unsatisfactory results in both QPPCaco and QPlogBB metrics.

To further refine the selection of potential drug candidates, MM-GBSA analysis was performed to evaluate the energetic contributions of ligand–enzyme complexes. As reported in Table 3, this analysis provides insights into the stability and binding affinity of each complex by calculating free binding energies (ΔG_bind_). Among the tested compounds, Nebivolol displayed the lowest ΔG_bind_ value, indicating a potentially stronger interaction with the enzyme. Overall, all compounds showed favorable ΔG_bind_ values, suggesting that they are likely to bind effectively. These findings offer critical insights into the stability and efficacy of the compounds, helping to guide the selection of candidates for further experimental validation.

Based on the analyses, five drugs, including Nebivolol, Dapagliflozin, Carvedilol, Floctafenine, and Benazepril, were selected for further investigation. For sodium–glucose transport protein 2 (SGLT2) inhibitors, including Canagliflozin, Dapagliflozin, and Ertugliflozin [54], a representative compound was chosen following a comprehensive review of the literature and the GCS–drug interactions outlined in Table 1. Notably, Dapagliflozin has been reported in the literature to exhibit significantly higher selectivity for SGLT2 over SGLT1, particularly intestinal SGLT1, compared to Canagliflozin [55]. This greater selectivity could help reduce drug-related side effects. Furthermore, Dapagliflozin shows a higher number of favorable interactions with GCS compared to Canagliflozin and Ertugliflozin, which may enhance its binding affinity. Based on these factors, Dapagliflozin was selected as the representative SGLT2 inhibitor. To assess the binding stability of the five selected drugs, 100 ns MD simulations were performed for each GCS–drug complex.

### 2.5. Binding Stability of Selected Drugs Through MD Simulations

**Figure 6:**
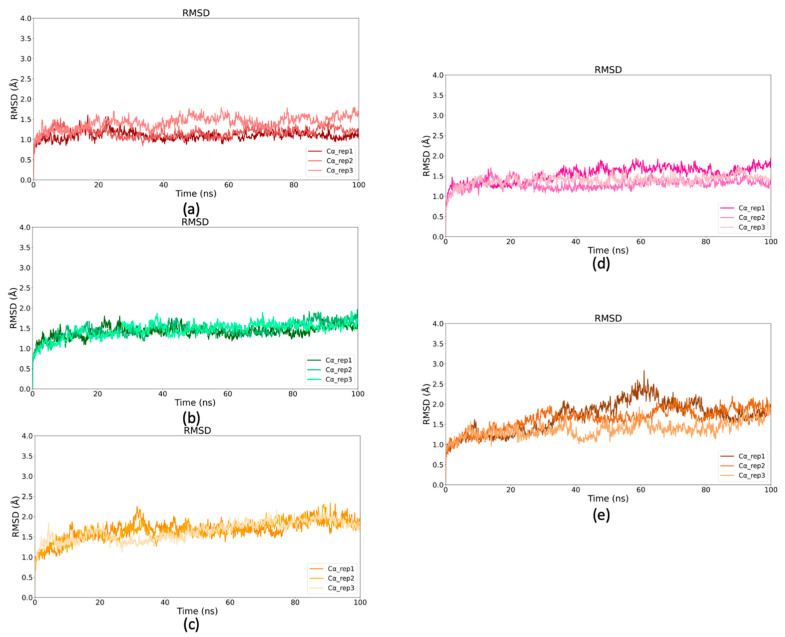
RMSD profiles of Cα atoms of GCS–ligand complexes. (**a**) RMSD profile of Cα atoms for GCS–Dapagliflozin complex. (**b**) RMSD profile of Cα atoms for GCS–Nebivolol complex. (**c**) RMSD profile of Cα atoms in GCS–Benazepril complex (**d**) RMSD profile of Cα atoms for GCS–Floctafenine complex. (**e**) RMSD profile of Cα atoms for GCS–Carvedilol complex.

To further assess the stability of the selected GCS–drug complexes, MD simulations were conducted for 100 ns, and Root Mean square Deviation (RMSD) analysis was performed. The RMSD values of the Cα atoms were calculated for each GCS–ligand complex to evaluate the effect of ligand binding on the conformational stability of the GCS enzyme during the simulations, shown in Figure 6a–e. Using the initial structure as a reference, RMSD values were plotted over time. The results indicated that all complexes achieved stable conformations after approximately 20 ns, with most showing RMSD values below 2.5 Å, reflecting good structural stability. However, the Carvedilol complex exhibited slightly higher RMSD values, reaching up to 3.0 Å, indicating more pronounced conformational changes in comparison to the others. Despite this, the overall structure of the GCS enzyme remained stable across all ligand complexes.

**Figure 7:**
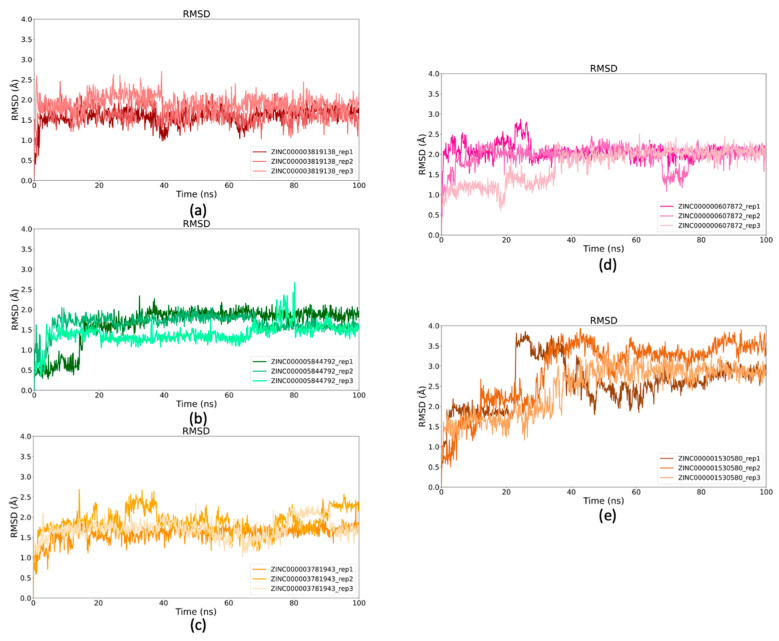
RMSD analysis of ligands over simulation time. (**a**) RMSD values of Dapagliflozin plotted against simulation time (ns). (**b**) RMSD of Nebivolol compared to the simulation period. (**c**) RMSD of Benazepril during the simulation. (**d**) RMSD of Floctafenine across the simulation time (**e**) RMSD of Carvedilol over the simulation duration.

RMSD values of the ligands were also analyzed to assess their stability within the active site. As shown in Figure 7a–e, Dapagliflozin reached significant stability early in the simulation, stabilizing around 1.5 Å, especially during the last 60 ns. Both Nebivolol and Benazepril reached stable conformations after the initial 20 ns, with Nebivolol maintaining RMSD values between 1 Å and 2 Å, while Benazepril exhibited similar stability, though one simulation reached an RMSD of 2.8 Å (Figure 7c). Floctafenine achieved stability after 40 ns, maintaining an RMSD of approximately 2 Å in the final 20 ns. Carvedilol, however, displayed higher RMSD values, plateauing only in the last 20 ns, with values between 2.5 Å and 4 Å, indicating lower stability compared to the other compounds.

Based on these computational analyses, Nebivolol (ZINC5844792), Dapagliflozin (ZINC3819138) and Benazepril (ZINC3781943) compounds were selected for in vitro testing, as their stability profiles within the active site suggest they are the most promising candidates for further experimental validation.

### 2.6. In Vitro Test

**Figure 8:**
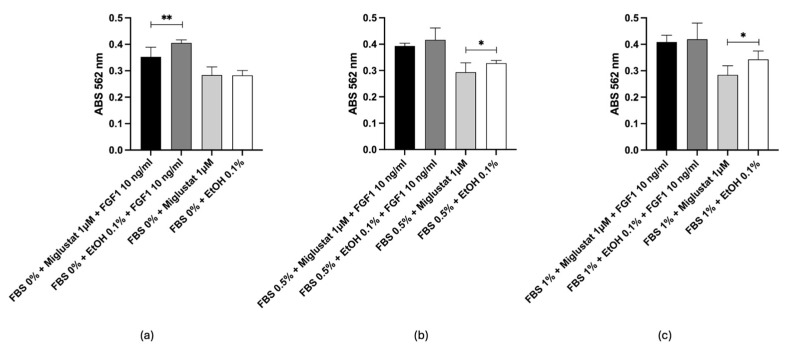
Miglustat in vitro test. (**a**) 3T3 Swiss fibroblast proliferation after treatment with Miglustat/EtOH ± FGF1 10 ng/mL with 0% FBS. (**b**) 0.5% FBS. (**c**) 1% FBS. * *p* < 0.05; ** *p* < 0.01.

The work by Li R. et al. [56] demonstrated that selective inhibition of GCS by D-l-threo-1-phenyl-2-hexadecanoylamino-3-pyrrolidino-1-propanol-HCl (PPPP) 1 µmol/L, without increasing ceramide levels, led to the inhibition of cellular ganglioside synthesis and blocked cell proliferation mediated by growth factors. This suggests that gangliosides present in cells promote growth factor-induced proliferation and that the interaction between cell surface gangliosides and growth factor receptors is essential for optimal fibroblast proliferation. As indicated in the study, it is hypothesized that depletion of cellular gangliosides disrupts the formation of glycosphingolipid clusters in the plasma membrane, which may be the mechanism underlying the blockade of growth factor-induced proliferation. Based on these findings, in vitro tests were conducted on 3T3 Swiss fibroblasts with FBS at different concentrations to verify the ability of the selected compounds to inhibit GCS. The results obtained with Miglustat were consistent with what is already known; in fact, it can be observed in Figure 8a that Miglustat significantly slowed down cell proliferation in the presence of the growth factor and in the absence of fetal bovine serum (FBS) compared to the same condition without Miglustat (*p* < 0.01). The drug did not induce cell death, as the absorbance of cells treated with Miglustat is comparable to that of untreated cells (Figure 8a). The presence of a low concentration of FBS (0.5% and 1%), in addition to the FGF1, allows cells to counterbalance the effect of Miglustat, which persists in the absence of FGF1 (Figure 8b,c respectively), confirming the drug’s ability to modulate cell proliferation by acting on enzymatic activity.

**Figure 9:**
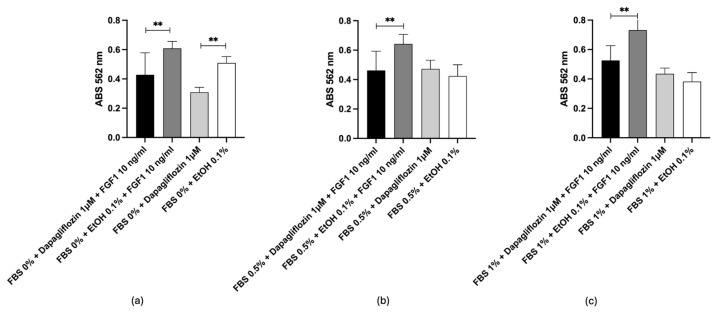
Dapagliflozin in vitro test. (**a**) 3T3 Swiss fibroblast proliferation after treatment with Dapagliflozin/EtOH ± FGF1 10 ng/mL with 0% FBS. (**b**) 0.5% FBS. (**c**) 1% FBS. ** *p* < 0.01.

Similar findings were observed with Dapagliflozin, as shown in Figure 9a–c. Also in this case, the drug inhibited proliferation without inducing cell death, acting specifically through drug–enzyme interactions, as confirmed by in silico analysis. In this case, the effect of Dapagliflozin is particularly evident in the presence of FGF1, regardless of the presence of FBS. Therefore, the results obtained indicate the need to verify whether Dapagliflozin, similarly to Miglustat, is an enzymatic inhibitor, as suggested by the in vitro results (Figure 8a–c).

With all the other compounds, no significant effects on proliferation were observed, as reported in Supplementary Materials (Nebivolol, Figure S4A–C and Benazepril Figure S5A–C). Therefore, further specific experiments will be needed to verify this hypothesis.

### 2.7. Comparative Binding Modes of Dapagliflozin and Miglustat

**Figure 10:**
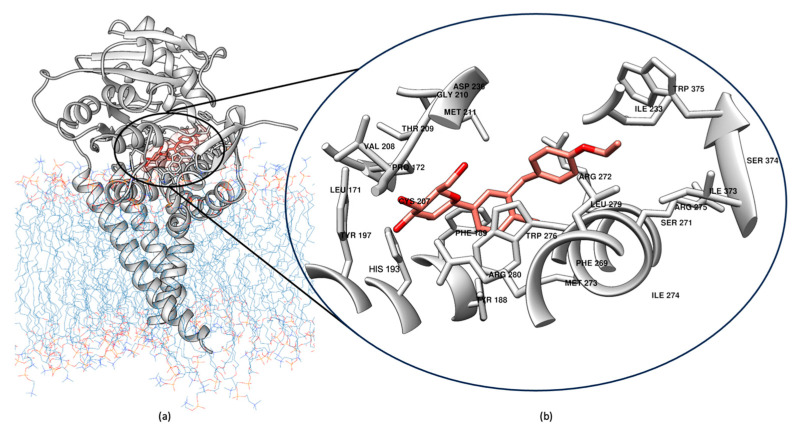
Close-up of the Dapagliflozin cluster, showed in salmon, after MD simulations. (**a**) The binding site of Dapagliflozin within the GCS active site, showing its interaction with key residues. (**b**) Close-up view highlighting the critical interactions between Dapagliflozin and GCS, including key amino acids involved in stabilizing the inhibitor.

After the successful in vitro inhibition of GCS activity by Dapagliflozin, a detailed structural analysis of its binding mode was performed to gain deeper insights into the molecular interactions underlying its inhibitory mechanism. Cluster analysis was applied to the combined trajectories from three 100 ns MD simulations, and the most representative structure of the GCS–Dapagliflozin complex was extracted. Figure 10 summarizes these findings: panel a shows the location of Dapagliflozin within the GCS binding site, while panel b provides a close-up view of the molecular interactions within a distance of 5 Å, which stabilize the inhibitor. As depicted in Figure 10b, Dapagliflozin interacts with residues critical for GCS activity. Specifically, the inhibitor establishes interactions with key amino acids, including His193, Cys207, Val208, Gly210, Asp236 (important for manganese coordination), and Trp276, which have been previously identified in the literature [49,53] and confirmed in earlier phases of this study [40] as crucial. These interactions, involving residues essential for substrate recognition and catalysis, reinforce Dapagliflozin’s mechanism of action as a GCS inhibitor.

**Figure 11:**
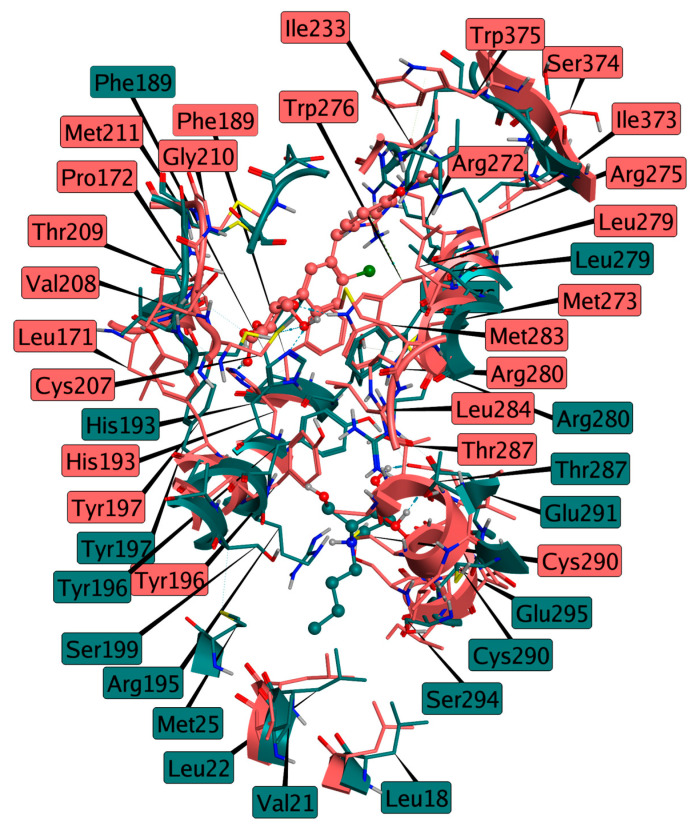
Superposition of the GCS–Miglustat complex (Miglustat in cyan, GCS structure in dark cyan, derived from the absolute minimum) and the GCS–Dapagliflozin complex (Dapagliflozin in salmon, GCS structure in rosy brown, derived from the cluster analysis), illustrating the distinct binding modes of the two inhibitors targeting different substrates of GCS.

Following the identification of Dapagliflozin as a potential GCS inhibitor mimicking UDP-glucose, Figure 11 presents a superposition of the absolute minimum structure of the GCS–Miglustat complex and the conformation derived from the cluster analysis of the GCS–Dapagliflozin complex. This comparison highlights the distinct binding modes of the two inhibitors, each targeting different substrates of GCS. Miglustat, acting as a ceramide mimic, binds to the GCS active site, interfering with the proper positioning of ceramide by obstructing the substrate’s access to the enzyme, specifically blocking its entrance from the membrane. In contrast, Dapagliflozin binds to the region of the active site typically occupied by UDP-glucose, further reinforcing its role as a UDP-glucose mimic. The structural comparison between these two complexes underscores the differing binding profiles of the inhibitors, suggesting that each targets a distinct functional aspect of GCS. While Miglustat competes for the ceramide binding site, Dapagliflozin interacts with the UDP-glucose binding site.

## 3. Materials and Methods

### 3.1. Induced Fit Docking for Miglustat, the Only Approved Drug for NPC

Induced fit docking [42,43,57] was performed for Miglustat using the Maestro Schrödinger software suite (v. 2020-3), with the docking grid centered on residues Asp236, Glu235, and His193. Two conditions were analyzed: one with Mn^2^⁺ in the active site and one without. The starting structure of the GCS enzyme was obtained from the lowest-energy configuration identified through metadynamics simulations, which determined the Mn^2^⁺ position within the active site [40]. Protein preparation was carried out using the Protein Preparation Wizard in Maestro Schrödinger software (v. 2020-3) with default settings. The induced fit docking workflow combined rigid receptor docking with Glide [42,43] and subsequent refinement of protein–ligand interactions using Prime [58,59]. Ligands were sourced from the PubChem database (PubChem CID for Miglustat: 51634) [60,61] and prepared using LigPrep, applying the OPLS3e force field [62]. Van der Waals scaling was set to 0.50 for both the receptor and ligand, and up to 20 poses of the ligand–protein complex were generated using standard precision (SP). The most favorable pose was selected for further MD simulations. During MD simulations, the presence of Mn^2^⁺ in the active site led to inhibitor dissociation. Therefore, only the results from induced fit docking and MD simulations without Mn^2^⁺ are reported.

### 3.2. MD Simulation of Miglustat

MD simulations were performed for Miglustat using GROMACS 2021.4 [63] software with the CHARMM36m [64] force field, which includes the WYF parameters to account for π–cation interactions [65]. The simulations were conducted at the atomistic level, employing the TIP3P water model [64]. System setup was facilitated through the CHARMM-GUI server [66] using the Membrane Builder tool, which was used to generate a phospholipid bilayer. The GCS protein was embedded in a 103-lipid POPC bilayer and solvated with 11,367 water molecules. To neutralize the system, K⁺ and Cl^−^ ions were added to achieve a 0.15 M salt concentration. All simulations were performed at a temperature of 303.15 K.

### 3.3. Well-Tempered FM Simulations of Miglustat

To determine the inhibitor’s position within the binding site and identify key amino acids involved in binding, a well-tempered FM analysis was conducted. The most representative GCS–inhibitor complex was selected from a cluster analysis of the final 500 ns of a 1 µs MD simulation. For the FM analysis, the biased CVs used were the position along the funnel’s rotation axis (CV1), the distance from the axis (CV2), and the distance between the carboxyl carbon of Asp236 and the nitrogen atom in the piperidine ring of Miglustat (CV3). The funnel parameters were set to Zcc = 3.8 nm, Alpha = 0.5 rad, and Rcyl = 0.3 nm. Gaussian functions with a height of 2 kJ/mol and a sigma value of 0.05 were applied every 1 ps. The initial configuration of the system is shown in Figure S1.

### 3.4. Cross-Docking Procedure

Cross-docking was performed using the XGlide [42,43], panel within the Maestro Schrödinger software suite (v. 2020-3), utilizing the xglide.py command-line script. Multiple receptor files and an input ligand file were specified in the parameter file to generate grids for different receptor conformations, followed by docking ligands to each receptor. Protein conformations were obtained from clustering results of MD simulations, comprising five poses each of GCS in its apo form and GCS bound to Miglustat. Additionally, five representative structures of GCS in the *holo* form (with endogenous substrates) were included, derived from FM simulations that captured the ceramide glycosylation transition state, studied in a previous work [40]. Ligands were selected from the ZINC database [41] in .mol2 format, which includes FDA-approved drugs and DrugBank-approved [52] compounds (n = 5903). Structural alignment of the receptor conformations was carried out using the utility structural alignment tool, with all atoms considered, and the first receptor configuration served as the reference structure. Receptor grids were generated with the docking box centered on the active site, defined by the coordinates of Asp236, Glu235, and His193, with a grid box radius of 26 Å to accommodate ligand flexibility. Ligands were prepared using LigPrep with the OPLS3e force field [62]. Cross-docking was conducted using the Glide module, in SP mode, and ligands with docking scores greater than 5 were further refined using the XP protocol [44]. Van der Waals scaling factors were set to 0.8 for nonpolar regions of both ligands and receptors to account for minor conformational changes. An RMSD cutoff of 2 Å was used to evaluate docking pose quality relative to reference ligands, with up to 20 poses generated per ligand. The top poses were analyzed based on their docking score, and key interactions, including H-bonds, hydrophobic contacts, and π-π stacking, were identified using the Maestro Schrödinger software (v. 2020-3) interface. Ligands with favorable binding affinity and interaction profiles were selected for subsequent VS.

### 3.5. Virtual Screening

The VS process was conducted using Glide [42,43,44], to refine potential drug candidates identified from cross-docking results. Initially, all ligands underwent evaluation using the Maestro Schrödinger software (v. 2020-3) QikProp module to assess their physicochemical properties and ADME profiles. Key parameters analyzed included the QPlogPo/w, QPPCaco, QPlogBB, molecular weight, and the number of H-bond donors and acceptors. Lipinski’s rule of five was applied to ensure drug-likeness, and compounds violating these criteria or containing reactive functional groups were excluded. A re-docking procedure was then performed using Glide’s XP mode, utilizing the same grid coordinates as in the cross-docking phase. Following this, the Prime MM-GBSA method was employed to predict the ΔG_bind_ of the top-ranked compounds from the Glide XP results. The ΔG_bind_ was calculated as

ΔG_bind_ = ΔG_solv_ + ΔE_MM_ + ΔG_SA_ (1)

where ΔG_solv_ represents the difference in GBSA solvation energy between the enzyme–ligand complex and the sum of solvation energies for the unbound protein and ligand [66,67], ΔE_MM_ is the difference in minimized molecular mechanics energies between the complex and the individual components, and ΔG_SA_ is the difference in surface area energies between the complex and the unbound states. The docking poses were further assessed by visual inspection, docking scores, and a review of potential side effects reported in the literature. The most promising compounds, based on these evaluations, were selected for further investigation through MD simulations.

### 3.6. Simulations of Top Ligands

MD simulations to evaluate the stability of protein–ligand complexes using GROMACS 2021.4 [63] software with the CHARMM36m [64] were performed. Each complex underwent 100 ns of MD simulation, with a total of 5 systems prepared. System setup was carried out using the CHARMM-GUI server [66], employing the Membrane Builder tool to generate a phospholipid bilayer. Each system was embedded within a POPC bilayer, and system neutrality was maintained by balancing the total charge with K⁺ and Cl^−^ ions, resulting in a 0.15 M salt concentration. Simulations were conducted at 303.15 K, with coordinates saved every 0.2 ps. Stability was assessed by analyzing the RMSD of the complexes to monitor conformational changes and overall structural stability.

### 3.7. In Vitro Tests

#### 3.7.1. Materials

Recombinant human fibroblast growth factor acidic (FGF1) from Sigma-Aldrich, Milan, Italy was stored as a stock solution at −20 °C at a concentration of 1 mg/mL. Miglustat, Nebivolol, Dapagliflozin and Benazepril were acquired from MedChemExpress (MCE), Princeton, NJ, USA. They were dissolved in ethanol (EtOH) to achieve a concentration of 1 mM and stored as stock solutions at −20 °C.

3T3 Swiss fibroblasts were kindly provided by Prof. Giovan Battista Pani, UCSC, Santa Cruz, CA, USA.

#### 3.7.2. Methods

Indirect assessment of cell proliferation following growth factors and cellular ganglioside depletion.

3T3 Swiss fibroblasts were seeded at 1 × 10^4^ cells/well in 96-well plates containing Dulbecco’s Modified Eagle Medium (DMEM), containing L-glutamine, HEPES buffer system, glucose at 4.8 g/L, phenol red, penicillin at 100 U/mL, streptomycin at 100 μg/mL, and 10% FBS, and incubated overnight. The cell culture medium was then replaced with fresh medium containing either 1 μmol/L of each drug or 0.1% (*v*/*v*) EtOH (as a solvent control), and the cells were cultured for 3 days. Subsequently, DMEM was removed and replaced with media containing 0% FBS or low FBS (0.5% or 1%) supplemented with either 1 μmol/L of each drug or 0.1% EtOH ± FGF1 10 ng/mL or only the drug/EtOH without FGF1. After an 18–24 h incubation period, cell viability was assessed using the 3-(4,5-dimethylthiazol-2-yl)-2,5-diphenyl-2H-tetrazolium bromide (MTT) assay. The MTT reagent was used to measure cell viability. The reduction of MTT leads to the formation of a violet-blue formazan precipitate, indicating viable cell metabolism. The MTT assay was conducted following the protocol described by Wataha et al. [67]. To each well, 20 μL of a 5 mg/mL MTT solution in phosphate buffered saline (PBS) was added to 200 μL of the culture medium. After a 4 h incubation at 37 °C, intracellular formazan crystals were dissolved in 200 μL of isopropanol containing 0.04 M HCl. The absorbance (Abs) of each well was measured at 562 nm using a ELx800 microplate reader (BioTek Ins, Agilent, Santa Clara, CA, USA). Each experiment was performed in triplicate, and results are expressed in terms of Abs.

## 4. Conclusions

Drug repurposing offers a promising pathway for developing therapies for LSDs, particularly sphingolipidoses, by leveraging existing drugs to expedite treatment availability and minimize adverse effects. A critical focus of this approach is understanding the binding mechanisms of inhibitors to GCS. The binding mode of Miglustat, a known ceramide analog [50] and GCS inhibitor, was thoroughly analyzed through induced fit docking, MD simulations, and free energy calculations. After 1901 ns of simulation, Miglustat was confirmed to stably bind at the ceramide-binding site, establishing crucial interactions with residues Arg280 and Trp276, as well as forming H-bonds with Glu291 and Glu295. These findings confirmed its mechanism of action, demonstrating that it inhibits ceramide glycosylation competitively with ceramide but non-competitively with UDP-glucose. A cross-docking approach was then employed to identify additional inhibitors from a library of 5903 compounds, including FDA-approved drugs. GCS conformations, derived from MD clustering and well-tempered FM simulations analysis, were screened using Glide docking techniques and refined based on docking scores, ADME profiles, and drug-likeness criteria. Subsequent MM-GBSA and MD simulations confirmed the binding stability of the selected candidates, identifying Dapagliflozin, Nebivolol, and Benazepril as the most promising options. The next step in this drug repurposing protocol was to estimate the capacity of the selected drugs in comparison with Miglustat, to interfere with the ceramide metabolic pathway. As demonstrated by a previous paper [56], a proliferation assay can indicate indirectly the depletion of cellular gangliosides. In this work, using the MTT assay, we were able to see that cells treated with effective compounds exhibited lower metabolic activity compared to untreated cells, as GCS inhibition prevents the formation of glycosphingolipid clusters, necessary for optimal interaction between growth factor receptors and the cell membrane. This effect resulted in a reduced production of formazan, as confirmed by absorbance decrease, which indicates reduced cell proliferation, confirming that ganglioside depletion impairs the response to growth factors and consequently hinders cell growth. Only Dapagliflozin shows an activity comparable to Miglustat. Notably, Dapagliflozin acts as a UDP-glucose analog, targeting a complementary mechanism to Miglustat, which functions as a ceramide analog [50]. This dual targeting strategy offers the potential for broader and more effective therapeutic options. The complementary mechanisms of action of Miglustat and Dapagliflozin underscore the value of drug repurposing in expanding the therapeutic strategies for LSDs. Further experimental validations, including in vivo testing and clinical studies, are essential to translate these findings into viable therapeutic options, with the ultimate goal of improving outcomes for patients with sphingolipidoses. In particular, it will be crucial in the future to assess any new interactions between the drug and the target disease that may arise from its use in a therapeutic indication different from the original. Another key aspect will be the adjustment of dosing regimens, as the optimal dose for repurposed drugs may differ significantly from that originally approved for the condition they were first intended to treat. These insights will ensure that the drug is both effective and safe for the target population. Continuous monitoring of patient responses, along with in-depth pharmacokinetic studies, will also be critical for optimizing these parameters and supporting the development of personalized treatment protocols, ultimately expanding therapeutic options for patients.

## Figures and Tables

**Table 1:** Re-docking XP results for selected molecules. This table reports the molecular name, ZINC code, docking score, and types of interactions (H-bonds, salt bridges, π-stacking) for each compound, along with the specific residues involved. The data reflects results obtained from the re-docking phase following initial cross-docking screenings.

Drugs	Zinc Code	Docking Score	Interactions	Residue
**Labetalol**	ZINC416	−11.769	H-bonds/ Salt bridge/ π-stacking	Asp144, Ile233, Glu235, Asp236, Arg275/Asp144, Glu235, Asp236, Arg275/Trp276
**Pitavastatin**	ZINC1534965	−11.305	H-bonds/ Salt bridge/ π-stacking	Lys124, Glu235, Asp236, Arg275, Trp375/Trp276
**Macimorelin**	ZINC1554197	−11.081	H-bonds/ Salt bridge/ π-stacking	Asp144, Ile233, Glu235, Asp236, Trp276/Asp144, Glu235, Asp236/Trp276
**Pravastatin**	ZINC3798763	−10.946	H-bonds/ Salt bridge	Gly210, Glu235, Asp236, Trp276, Arg280/Lys124
**Ertugliflozin**	ZINC68197809	−10.525	H-bonds/ π-stacking	His193, Glu291/Trp276
**Canagliflozin**	ZINC43207238	−10.244	H-bonds	His193, Thr209, Gly210
**Nebivolol**	ZINC5844792	−10.153	H-bonds/ Salt bridge/π-stacking	Asp144, Asp236/Asp144, Glu235, Asp236/Trp276
**Dapagliflozin**	ZINC3819138	−10.138	H-bonds	His193, Thr209, Val208, Asp236, Arg275
**Benazepril**	ZINC3781943	−10.456	H-bonds/ π-stacking	Asp236, Glu235, Gly210/Trp375
**Floctafenine**	ZINC607872	−9.241	H-bonds/ π-stacking	His193, Glu291/Trp276
**Carvedilol**	ZINC1530580	−9.142	H-bonds/ Salt bridge/ π-stacking	Asp144, Asp 236/Asp144, Glu235, Asp236/Trp276

**Table 2:** Lipinski’s rule evaluation for in silico drug candidates. This table presents data for each compound, including the compound name, ZINC code, number of H-bonds donors, number of H-bonds acceptors, molecular weight, and QPlogPo/w.

Drugs	Zinc Code	HB Donor	H-Bonds Donor Acceptor	Rule of Five	QPlogPo/w	Molecular Weight
**Nebivolol**	ZINC5844792	3	6.4	0	3.826	405.441
**Canagliflozin**	ZINC43207238	4	8.5	0	3.249	444.517
**Dapagliflozin**	ZINC3819138	4	9.250	0	2.236	408.878
**Carvedilol**	ZINC1530580	3	5.450	0	4.208	406.480
**Ertugliflozin**	ZINC68197809	4	9.050	0	2.458	436.888
**Floctafenine**	ZINC607872	2	5.9	0	3.971	406.361
**Benazepril**	ZINC3781943	2	8.5	0	1.715	424.496
**Pravastatin**	ZINC3798763	3	8.1	0	3.351	424.533
**Pitavastatin**	ZINC1534965	2	5.4	0	4.858	421.467
**Labetalol**	ZINC416	4	5.450	0	2.874	328.410

**Table 3:** Table of MM-GBSA binding free energies for compounds. This table provides the molecular name, ZINC code, and MM-GBSA calculated ΔG_bind_ for each compound. ΔG_bind_ values provide details on the strength of interaction between the compounds and the GCS enzyme, highlighting their potential efficacy as inhibitors.

Drugs	Zinc Code	MM-GBSA (ΔG_bind_)
**Nebivolol**	ZINC5844792	−67.8
**Canagliflozin**	ZINC43207238	−64.5
**Dapagliflozin**	ZINC3819138	−63.93
**Carvedilol**	ZINC1530580	−63.51
**Ertugliflozin**	ZINC68197809	−57.18
**Floctafenine**	ZINC607872	−53.58
**Benazepril**	ZINC3781943	−39.39

## Data Availability

The raw data supporting the conclusions of this article will be made available by the authors on request.

## Supplementary Materials

The supporting information can be downloaded at https://www.mdpi.com/article/10.3390/ijms26052195/s1.

## Abbreviations

The following abbreviations are used in this manuscript:

LSDs	Lysosomal storage diseases
GCS	Glucosylceramide synthase
BMT	Bone marrow transplantation
ERT	Enzyme replacement therapy
SRT	Substrate reduction therapy
OCT2	Organic cation transporter 2
Mn2+	Manganese ion
MD	Molecular dynamics
FM	Funnel metadynamics
CVs	Collective variables
dCG-N	Distance between the carbon atom of the carboxyl group in Asp236 and the nitrogen atom in Miglustat’s piperidine ring
Cα atoms	Alpha-carbons
H-bonds	Hydrogen bonds
SP	Standard precision
XP	Extra precision
VS	Virtual screening
QPPCaco	Predicted apparent Caco-2 cell permeability in nm/sec
QPlogBB	Predicted brain/blood partition coefficient
QPlogPo/w	Predicted octanol/water partition coefficient
CNS	Central nervous system
PSA	Polar surface area
MM-GBSA	Molecular mechanics/generalized born surface area
ΔGbind	Free energy
SGLT1	Sodium-glucose transport protein 1
SGLT2	Sodium-glucose transport protein 2
RMSD	Root Mean square Deviation
PPPP	D-l-threo-1-phenyl-2-hexadecanoylamino-3-pyrrolidino-1-propanol-HCl
FBS	Fetal bovine serum
FGF1	Recombinant human fibroblast growth Factor acidic
MCE	MedChemExpress
EtOH	Ethanol
DMEM	Dulbecco’s Modified Eagle Medium
MTT	3-(4,5-dimethylthiazol-2-yl)-2,5-diphenyl-2H-tetrazolium bromide
PBS	Phosphate buffered saline
Abs	Absorbance
