# Supplementary material for: Targeting Glucosylceramide Synthase: Innovative Drug Repurposing Strategies for Lysosomal Diseases
Source: Int J Mol Sci. 2025 Feb 28;26(5):2195. doi: 10.3390/ijms26052195 (PMC11900012; doi:10.3390/ijms26052195)
Supplement: Supplementary file 1 [file ijms-26-02195-s001.zip › ijms-3450970-supplementary.pdf]

Supplementary Materials

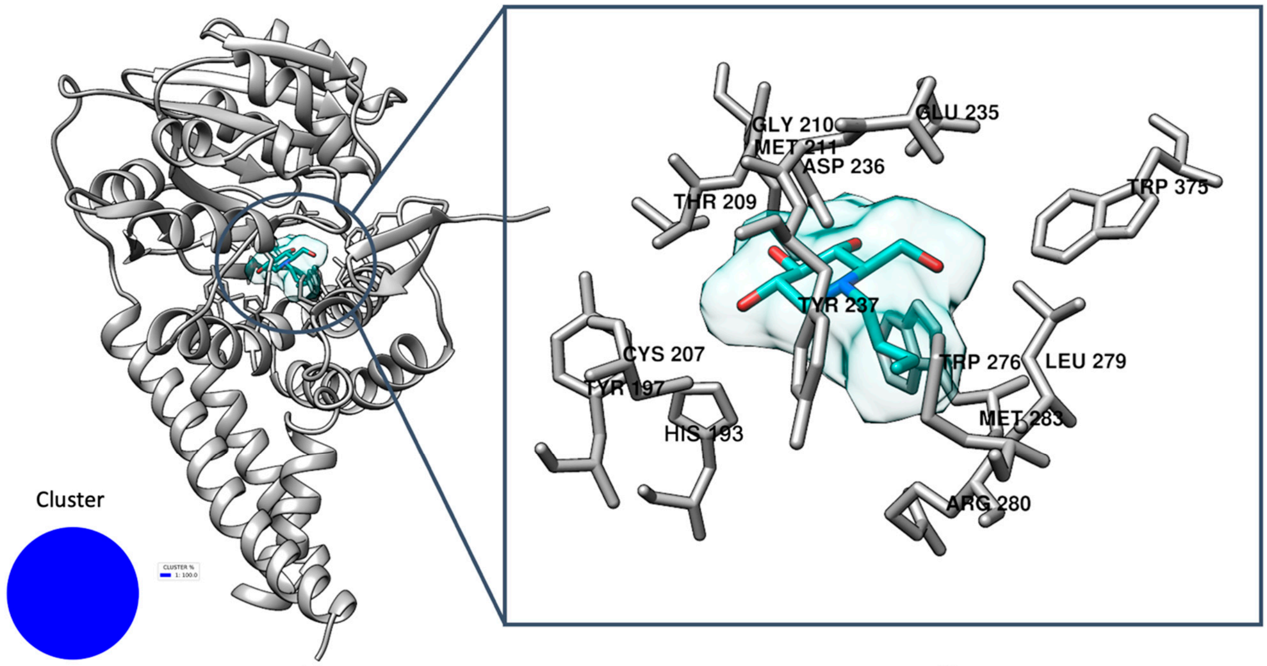

(a) (b)

Figure S1. Representative conformations of the GCS-Miglustat complex. (a) Cluster analysis of the final 500 ns of Molecular Dynamics (MD) simulation [1-3], highlighting a single representative cluster. (b) Close-up of amino acids involved in inhibitor interactions within a 5 Å distance.

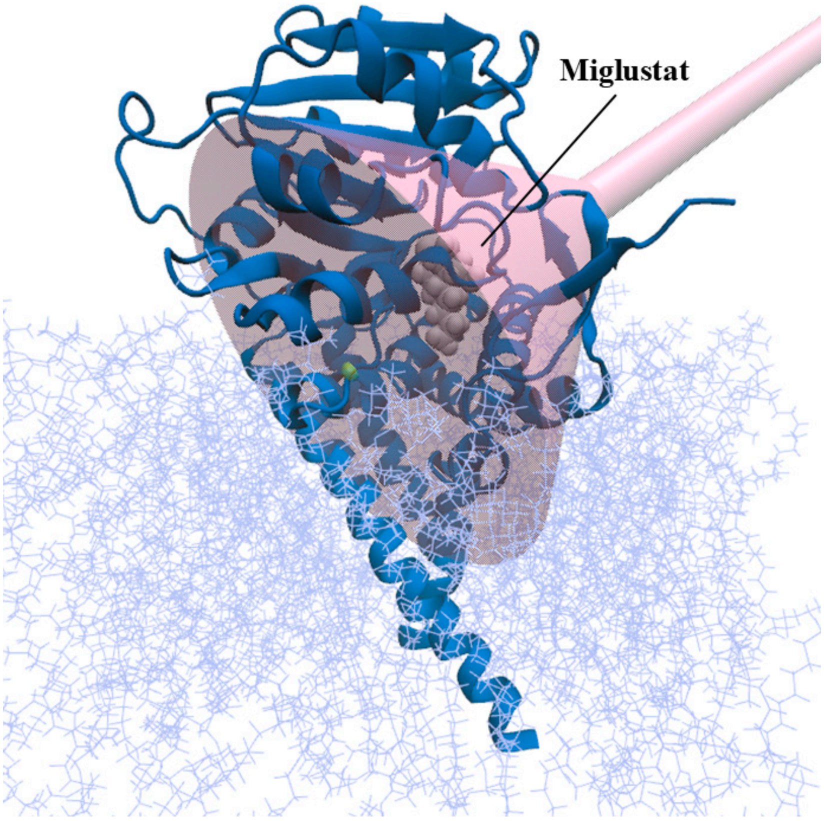

Figure S2. Initial configuration of the well-tempered Funnel Metadynamics (FM) system used to examine the position and conformation of the GCS inhibitor. The inhibitor is shown as gray spheres, while the membrane is represented in blue.

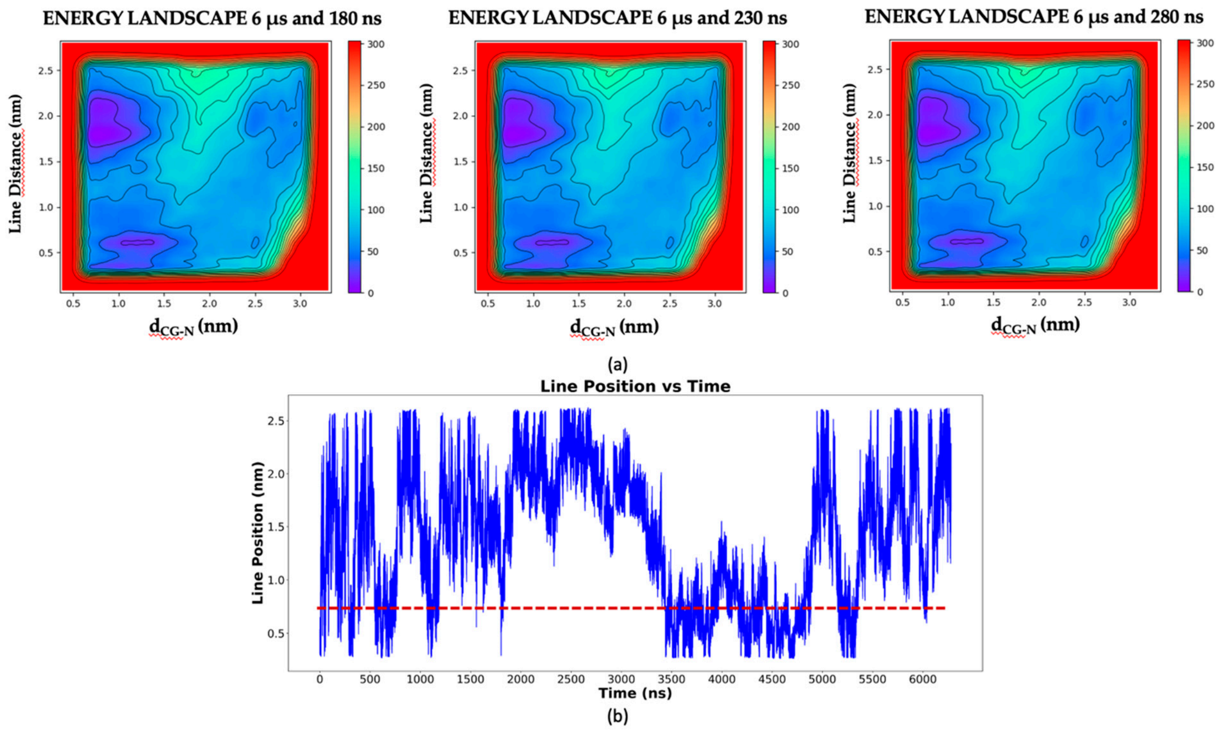

Figure S3. FM analysis. (a) Energy landscapes at different time intervals, each graph showing a 50 ns progression, demonstrating the convergence of the FM simulation. (b) During the simulation, Miglustat frequently occupies the ceramide binding site. The specific time when Miglustat aligns with the ceramide position are indicated by a red dashed line, highlighting critical interaction points.

Receptors	n°Molecules
GCS apo 1	3662
GCS apo 2	3938
GCS apo 3	4122
GCS apo 4	4131
GCS apo 5	3636
GCS holo 1	8851

GCS holo 2	8833
GCS holo 3	8843
GCS holo 4	8844
GCS holo 5	8844
GCS inhibitor 1	6345
GCS inhibitor 2	6349
GCS inhibitor 3	6352
GCS inhibitor 4	6347
GCS inhibitor 5	6346

Table S1 The table provides the number of ligands docked for each GCS conformation using the Glide XP protocol [4] for Cross-Docking [5, 6]. It details the quantity of ligands successfully docked across various receptor conformations analyzed with the Glide XP docking procedure.

Receptors	n° Molecules
GCS apo 1	237
GCS apo 2	194
GCS apo 3	202

GCS apo 4	202
GCS apo 5	183
GCS holo 1	202
GCS holo 2	188
GCS holo 3	41
GCS holo 4	56
GCS holo 5	190
GCS inhibitor 1	74
GCS inhibitor 2	148
GCS inhibitor 3	42
GCS inhibitor 4	100
GCS inhibitor 5	115

Table S2. Number of ligands docked for each GCS conformation using the Glide XP Re-Docking protocol in virtual screening (VS) workflow [5, 6, 8], with additional evaluations using the QikProp tool and Lipinski's rule. This table shows the number of ligands successfully docked to different conformations of the GCS enzyme.

Drugs	Zinc Code	QPPCaco	QPlogBB
Nebivolol	ZINC5844792	384.195	-0.210

Macimorelin	ZINC1554197	7.722	-1.921
Canagliflozin	ZINC43207238	349.322	-1.281
Dapagliflozin	ZINC3819138	234.485	-1.555
Carvedilol	ZINC1530580	493.818	-0.549
Ertugliflozin	ZINC68197809	317.516	-1.463
Floctafenine	ZINC607872	458.627	-1.147
Benazepril	ZINC3781943	18.688	-1.299
Pravastatin	ZINC3798763	16.672	-2.561
Pitavastatin	ZINC1534965	56.069	-1.570
Labetalol	ZINC416	33.022	-1.722

Table S3. *In Silico* ® DME Predictions for Potential Drug Molecules. This table presents the compound name, ZINC [7] code, QPPCaco (apparent permeability in the Caco-2 cell assay) values, and QPlogBB (prediction of blood-brain barrier permeability) values for each compound.

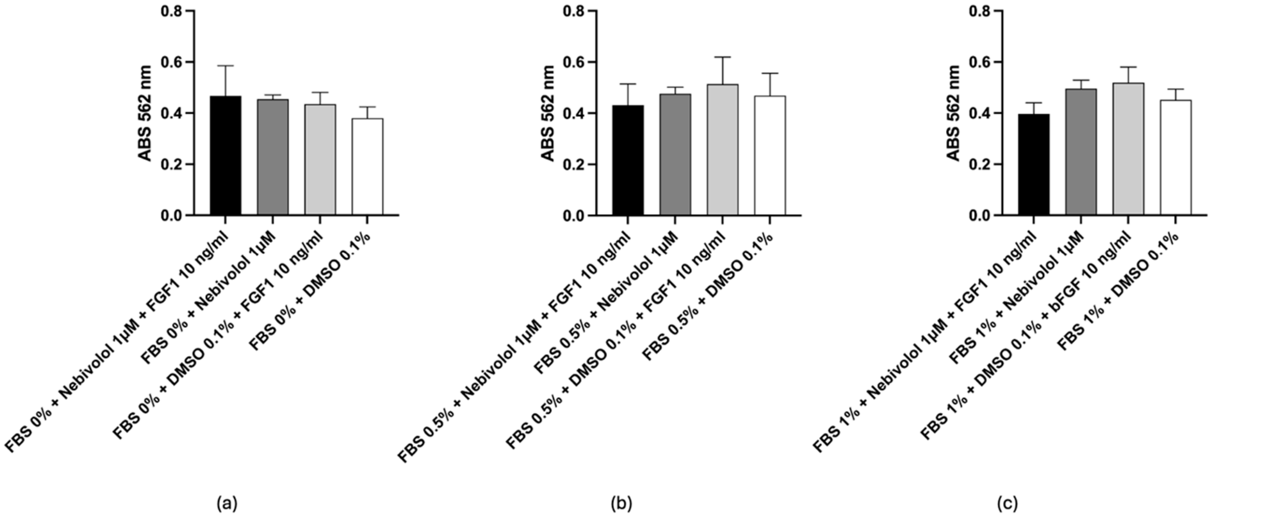

Figure S4. Nebivolol in vitro test. (a) 3T3 Swiss Fibroblasts proliferation after treatment with Nebivolol/EtOH ± FGF1 10 ng/ml with 0% FBS. (b) 0.5% FBS (c) 1% FBS. . * $p < 0.05$; ** $p < 0.01$; *** $p < 0.001$

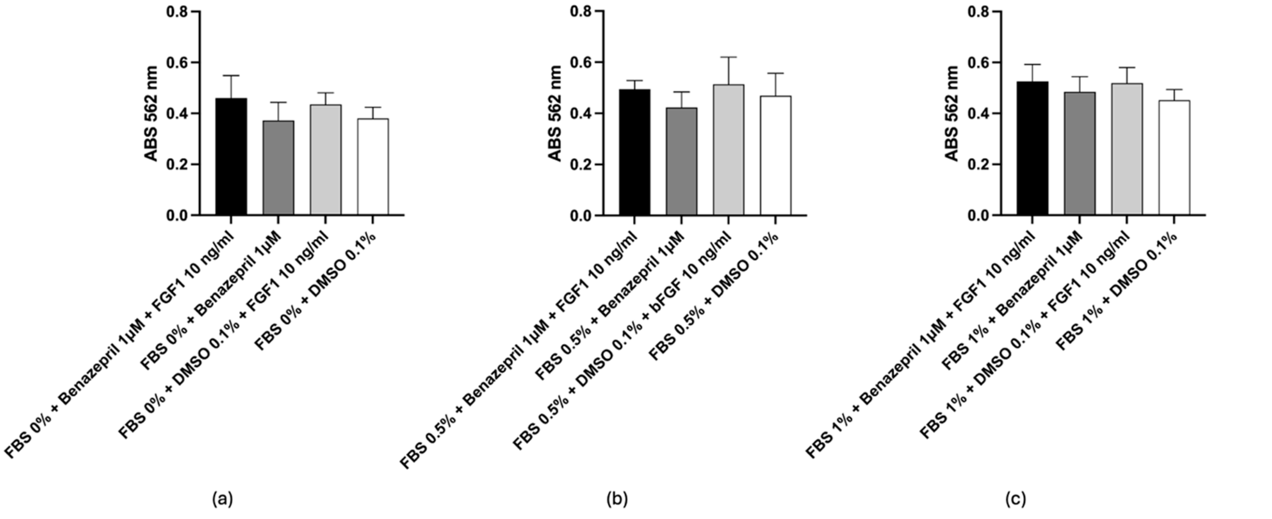

Figure S5. Benazepril in vitro test. (a) 3T3 Swiss Fibroblasts proliferation after treatment with Benazepril/EtOH ± FGF1 10 ng/ml with 0% FBS. (b) 0.5% FBS (c) 1% FBS. . * $p < 0.05$; ** $p < 0.01$; *** $p < 0.001$

References:

1. M. J. ◎ braham *et al.*, "GROM◎ CS: High performance molecular simulations through multi-level parallelism from laptops to supercomputers," *SoftwareX*, vol. 1–2, pp. 19–25, Sep. 2015, doi: 10.1016/j.softx.2015.06.001.
2. J. Huang *et al.*, "CH◎ RMM36m: an improved force field for folded and intrinsically disordered proteins," *Nat Methods*, vol. 14, no. 1, pp. 71–73, Jan. 2017, doi: 10.1038/nmeth.4067.
3. H. M. Khan, ◎ . D. MacKerell, and N. Reuter, "Cation-π Interactions between Methylated ◎ mmonium Groups and Tryptophan in the CH◎ RMM36 ◎ dditive Force Field," *J Chem Theory Comput*, vol. 15, no. 1, pp. 7–12, Jan. 2019, doi: 10.1021/acs.jctc.8b00839.
4. R. ◎ . Friesner *et al.*, "Extra Precision Glide: Docking and Scoring Incorporating a Model of Hydrophobic Enclosure for Protein−Ligand Complexes," *J Med Chem*, vol. 49, no. 21, pp. 6177–6196, Oct. 2006, doi: 10.1021/jm051256o.
5. R. ◎ . Friesner et al., "Glide: ◎ New ◎ pproach for Rapid, ◎ ccurate Docking and Scoring. 1. Method and ◎ ssessment of Docking ◎ ccuracy," J Med Chem, vol. 47, no. 7, pp. 1739–1749, Mar. 2004, doi: 10.1021/jm0306430.
6. T. ◎ . Halgren et al., "Glide: ◎ New ◎ pproach for Rapid, ◎ ccurate Docking and Scoring. 2. Enrichment Factors in Database Screening," J Med Chem, vol. 47, no. 7, pp. 1750–1759, Mar. 2004, doi: 10.1021/jm030644s.
7. T. Sterling and J. J. Irwin, "ZINC 15 – Ligand Discovery for Everyone," J Chem Inf Model, vol. 55, no. 11, pp. 2324–2337, Nov. 2015, doi: 10.1021/acs.jcim.5b00559.
8. R. ◎ . Friesner *et al.*, "Extra Precision Glide: Docking and Scoring Incorporating a Model of Hydrophobic Enclosure for Protein−Ligand Complexes," *J Med Chem*, vol. 49, no. 21, pp. 6177–6196, Oct. 2006, doi: 10.1021/jm051256o.
